# Concanavalin A Delivers a Photoactive Protein to the Bacterial Wall

**DOI:** 10.3390/ijms25115751

**Published:** 2024-05-25

**Authors:** Andrea Mussini, Pietro Delcanale, Melissa Berni, Stefano Pongolini, Mireia Jordà-Redondo, Montserrat Agut, Peter J. Steinbach, Santi Nonell, Stefania Abbruzzetti, Cristiano Viappiani

**Affiliations:** 1 Dipartimento di Scienze Matematiche, Fisiche e Informatiche, Università di Parma, Parco Area delle Scienze 7A, 43124 Parma, Italy; 2 Institut Químic de Sarrià, Universitat Ramon Llull, Via Augusta 390, 08017 Barcelona, Spain; 3 Risk Analysis and Genomic Epidemiology Unit, Istituto Zooprofilattico Sperimentale della Lombardia e dell’Emilia-Romagna, Strada dei Mercati, 13/A, 43126 Parma, Italy; 4 Bioinformatics and Computational Biosciences Branch, National Institute of Allergy and Infectious Diseases, National Institutes of Health, Bethesda, MD 20892, USA

**Keywords:** photodynamic effect, targeted photodynamic inactivation, single molecule localization microscopy, dSTORM, photosensitizer, fluorescence correlation spectroscopy, diffusion times distribution

## Abstract

Modular supramolecular complexes, where different proteins are assembled to gather targeting capability and photofunctional properties within the same structures, are of special interest for bacterial photodynamic inactivation, given their inherent biocompatibility and flexibility. We have recently proposed one such structure, exploiting the tetrameric bacterial protein streptavidin as the main building block, to target *S. aureus* protein A. To expand the palette of targets, we have linked biotinylated Concanavalin A, a sugar-binding protein, to a methylene blue-labelled streptavidin. By applying a combination of spectroscopy and microscopy, we demonstrate the binding of Concanavalin A to the walls of Gram-positive *S. aureus* and Gram-negative *E. coli*. Photoinactivation is observed for both bacterial strains in the low micromolar range, although the moderate affinity for the molecular targets and the low singlet oxygen yields limit the overall efficiency. Finally, we apply a maximum entropy method to the analysis of autocorrelation traces, which proves particularly useful when interpreting signals measured for diffusing systems heterogeneous in size, such as fluorescent species bound to bacteria.

## 1. Introduction

Due to the excessive and incorrect use of antibiotics, antimicrobial resistance [1] appears to be the major emerging global health threat for the coming decades [2]. The decreasing number of available effective antimicrobials and the insufficient rate of development of new replacements [3] call for investigations of alternative methodologies that are immune to resistance [4]. Among them, antimicrobial photodynamic inactivation (PDI) is receiving renewed interest [5,6,7,8,9,10]. The principle of PDI is quite simple: utilizing a photoactive molecule, or photosensitizer (PS), that, upon absorption of visible light, undergoes intersystem crossing to a triplet state in appreciable yield. In this state, reactive oxygen species (ROS) and/or singlet oxygen are produced that result in light-induced bacterial cell toxicity. The short lifetime of the photoinduced reactive oxygen species (ca. 3 μs for singlet oxygen [11,12,13,14]) has important consequences for the design of effective photosensitizers. It has been estimated that the distance singlet oxygen can diffuse before spontaneous deexcitation is on the order of 200 nm [15]. This implies that, in order to be effective, the photoactive compound must be in close proximity to the molecular target on the bacterial structure. Although the molecular species affected by photooxidation comprise proteins, lipids, and nucleic acids [16], it is generally accepted that bacterial phototoxicity mostly results from damage at the level of cytoplasmic membranes and DNA [17,18]. With the aim of providing PSs with some targeting ability, positively charged molecules have been devised that exploit the strong electrostatic interactions with the negatively charged bacterial wall, composed by the phospholipidic bilayer and peptidoglycans, respectively rich in phosphate and hydroxyl groups. To this purpose, PSs functionalized with small cationic functional groups have been developed [19,20,21,22]. Cationic PSs such as methylene blue [23] and other phenothiazinium derivatives [24], or cationic porphyrins [25] and phthalocyanines [26] have been successfully employed. Recent approaches to targeted delivery in antimicrobial PDI have been reviewed [6].

Among the carriers explored for the delivery of photosensitive compounds to their targets [7,27,28], proteins hold a special place due to their natural biocompatibility. Water-soluble proteins with hydrophobic cavities can be adopted as delivery systems for water-insoluble photosensitizers, increasing their solubility and bioavailability [29]. These systems are devoid of target specificity and act as passive carriers, facilitating the diffusion of the photosensitizer to cellular components, mostly membranes, to which the photosensitizer is downloaded. A few attempts have been made to introduce targeting capability in the delivery systems, through the conjugation of the PS to, e.g., antibiotics or antibodies [6,30,31,32,33,34,35,36].

We have recently proposed a modular supramolecular construct around the the tetrameric protein streptavidin (strep) [37]. Strep was covalently labeled with eosin to make it capable of photosensitizing singlet oxygen and was linked to a biotinylated IgG so that *S. aureus* protein A could be targeted. As a result, the photoactive conjugate binds *S. aureus*, and induces efficient photoinactivation of this bacterial strain at sub-μM concentrations.

Thanks to the high affinity and specificity of the interactions, exploiting protein-protein interactions for targeting specific microbial constituents is a powerful approach, but it may be subject to the development of resistance mechanisms upon mutation of the targeted bacterial protein.

**Figure 1:**
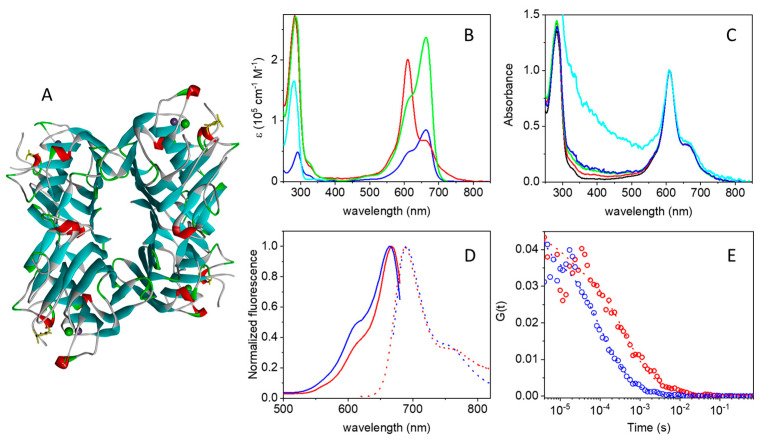
(**A**) Cartoon representation of the three-dimensional structure of the tetrameric Concanavalin A (PDB 3qlq). Ca^2+^ and Mn^2+^ ions are represented as solid spheres. (**B**) Molar absorption spectra for strep (cyan), MB (blue), and MB-strep (red). The green curve is obtained as a linear combination of the strep and MB spectra, where the MB spectrum was multiplied by 2.8. (**C**) Normalized absorption spectra (at 610 nm) for MB-strep in PBS as a function of protein concentration: black 4.6 µM, red 1.1 µM, green 0.55 µM, blue 0.25 µM, cyan 0.175 µM. Although the spectrum collected at the lowest concentration is strongly affected by scattering, the proportion of the two peaks (610 nm vs. 663 nm) in the visible band is similar to that observed at higher concentrations. (**D**) Peak normalized fluorescence excitation (solid lines) and emission (dotted lines) for MB (blue) and MB-strep (red) in PBS buffer. Fluorescence emission was collected with excitation at 655 nm for MB and 610 nm for MB-strep. Fluorescence excitation was collected with emission at 710 nm for MB and 690 nm for MB-strep. (**E**) Cross correlation curves for MB (blue circles) and MB-strep (red circles), fit by a single diffusing species (dotted lines). Excitation at 635 nm, detection through a 650–700 nm emission filter. All experiments were performed in PBS buffer, pH = 7.4.

The sugar layer decorating the bacterial wall may be less prone to the development of resistance and represents an interesting expansion of the palette of targets for antimicrobial PDI. Cantelli et al. recently reported that by conjugating the well-characterized photosensitizer Rose Bengal with Concanavalin A (ConA), they were able to obtain an effective compound for targeted photodynamic therapy against Gram-negative bacteria [38]. ConA, originally extracted from the jack-bean (*Canavalia ensiformis*), is a homotetramer at neutral pH with a molecular weight of about 106 kDa (Figure 1A). Its molar absorbance coefficient at 280 nm is 116,000 M^−1^cm^−1^ [39]. Its structure is mostly formed by β strands [40]. Each monomer coordinates Ca^2+^ and Mn^2+^ ions. ConA belongs to the lectin family proteins characterized by specificity for binding polysaccharides containing α-glucose, mannose, glucosamine, and α-N-acetylglucosamine [41]. Glycans are present on the surface of all types of cells and play roles in the immune system, cellular signaling, and host-microbe interactions [42]. Given their affinity for carbohydrates, lectins bind successfully to cellular membranes, but their adherence varies accordingly to the diversity and number of saccharides found on them [43]. These proteins have shallow but well-established ligand-binding sites to recognize one or a few moieties of oligosaccharides and do not present any catalytic activity [44]. ConA has a dissociation constant of $K_{d}$ = 0.25 µM for glycogen and 2.89 µM for mannan [45]. In Gram-negative bacteria, ConA can bind the glucose and mannose residues contained in lipopolysaccharides [38]. Moreover, ConA can bind teichoic acids in Gram-positive bacteria since they are made up of α-glycosylates (e.g., in *S. epidermidis*) and α-N-acetylglucosamine (e.g., in *S. aureus*) [41,46].

In this work, we elaborate on the modular construct based on streptavidin and show that by replacing the targeting unit with ConA in the supramolecular complex, it is possible to drive the photoactive compound to saccharides on the bacterial wall. A commercial conjugate of Strep with the well-characterized photosensitizer methylene blue (MB) [8,47] was linked to biotinylated ConA. Importantly, we show that the supramolecular construct is effective in antimicrobial PDI against Gram-negative *E. coli* and Gram-positive *S. aureus*.

## 2. Results and Discussion

### 2.1. Photophysics of the MB-Streptavidin Conjugate

The absorption spectra of strep, MB, and the commercial conjugate MB-strep are compared in Figure 1B, where the molar absorption coefficients for the three species are reported. The molar absorption coefficients of MB (blue curve) and strep (cyan curve) are taken from the literature [48,49], whereas for MB-strep (red curve), ε was calculated from the measured absorption spectrum for a MB-strep solution of known molar concentration.

The change in shape of the visible absorption spectrum of the conjugated dye is evident, possibly due to the interaction of MB with protein residues and/or different MB molecules bound to the same streptavidin tetramer. In particular, the main absorption peak in the red is located at 663 nm with a shoulder at ≈610 nm for dilute MB aqueous solutions at pH 7.4. When bound to strep, the band at 610 nm becomes dominant, and the contribution at 663 nm becomes a shoulder of the main band (Figure 1B). Although the absorption spectrum of the MB dimer is similar to that reported in Figure 1A for MB-strep [47,48,50], an effect of MB dimerization between dyes linked to different streptavidin tetramers appears unlikely. The dimerization constant for MB was reported to be on the order of 10^3^ M^−1^ in water [48,51] and should lead to a very small fraction of dimers in the micromolar range. The presence of the protein linked to MB is expected to further reduce the dimerization constant. Importantly, the shape of the visible band in the absorption spectrum is independent of MB-strep conjugate concentration over a nearly 30-fold change in concentration, as shown in Figure 1C, which rules out intermolecular conjugate-conjugate interactions. A possible explanation is that the tetramer assembly brings MB molecules linked to different monomers in close proximity, thus favoring interactions similar to those experienced within MB dimers. The solvatochromic effects of protein-bound MB may partly contribute to the observed spectral changes [52].

The change in spectral shape of MB upon binding to strep prevents a reliable determination of the degree of labeling (DOL, defined as the number of bound dye molecules per strep monomer), since the molar absorption coefficient of the bound dye in the visible range is strongly affected by binding (Figure 1B, red curve). An estimate of the DOL can be obtained by assuming that the near-UV portion of the absorption spectrum of MB (wavelengths below 400 nm) is not markedly affected by binding. Under this assumption, the measured spectrum for the covalent adduct MB-strep (Figure 1B, red curve) can be reasonably reproduced by a linear combination of the spectra for strep and MB. The best result indicates that for each streptavidin tetramer, on average ca. 2.8 MB molecules are bound (Figure 1B, green curve), corresponding to a DOL ≈ 0.7 (i.e., ca. 0.7 MB molecules per strep monomer).

The interaction between bound MB and either amino acid residues or other copies of MB bound to the same streptavidin tetramer affects not only the absorption spectrum but also the fluorescence emission. Figure 1D compares normalized excitation and emission spectra for MB (blue) and the MB-streptavidin conjugate (red). The excitation spectra have comparable, although not identical, shapes, with a small red shift of the peak for MB-streptavidin (667 nm) in comparison to MB (663 nm). A comparison between the absorption (Figure 1B, red) and the fluorescence excitation spectra (Figure 1D, red) of the MB-streptavidin conjugate and the absorption spectrum of free MB shows that fluorescence emission of the conjugate is observed only from the free-like, i.e., non-interacting, MB-absorbing species. In short, MB is bound to at least two different classes of sites in the conjugate ensembles.

Fluorescence emission by the MB-strep conjugate is much weaker than that by MB, for which a quantum yield of *Φ_F_* = 0.04 was reported [47,53]. The fluorescence quantum yield of MB-strep in PBS was determined experimentally by a comparative method [54], using MB as a reference. In keeping with the coexistence of MB populations with distinct absorption spectra (Figure 1B), the value of *Φ_F_* for MB-strep was found to be dependent on excitation wavelength, being ca. 0.4 × 10^−3^ under 610 nm excitation and ca. 3 × 10^−3^ under 663 nm excitation. The very low emission by MB-strep is responsible for the high background in the near-infrared portion of the emission spectrum (red dotted curve in Figure 1C).

The integrity of the MB-strep complex can be checked through fluorescence anisotropy. Upon binding to strep, the anisotropy of MB emission increases from 0.09 to roughly 0.25, confirming that for the complex, rotational averaging is inhibited, as expected for a fluorophore bound to a large molecule like strep [54]. The short lifetime of the excited singlet state of MB is responsible for the relatively high anisotropy value of the free dye [54].

Additional evidence of the association between MB and streptavidin comes from fluorescence correlation spectroscopy (FCS). Due to the low fluorescence emission of MB and its MB-strep adduct, these fluorophores are admittedly less than ideal for FCS experiments. Nevertheless, using relatively high concentrations (≈μM), we were able to collect cross-correlation traces with a sufficiently high signal-to-noise ratio. The cross-correlation curve for MB (blue circles in Figure 1E) is best described by a single diffusing species with a diffusion coefficient of *D* = 220 ± 50 µm^2^/s. When MB is bound to strep (red circles in Figure 1E), diffusion becomes much slower, and the resulting cross-correlation curve can be fitted with a single diffusing species with *D* = 60 ± 10 µm^2^/s. In both cases, a triplet decay is observed in the microsecond time scale, but a quantitative analysis is made difficult by the low signal-to-noise ratio in this time range. Binding of MB-strep to ConA does not lead to changes in the diffusion coefficient that are detectable by FCS.

### 2.2. Photoactivity of MB-Streptavidin

**Figure 2:**
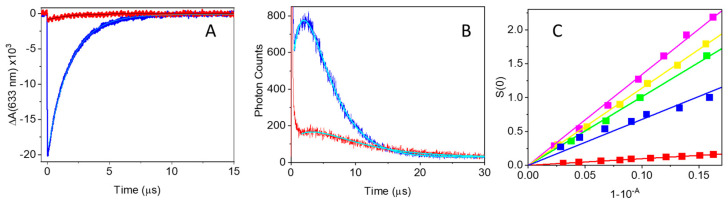
(**A**) Triplet state decay for MB (blue) and MB-strep (red) after excitation at 580 nm. Detection at 633 nm. PBS solutions were air-equilibrated. Cyan curves are the results of fits with single exponential decays. (**B**) Time-resolved phosphorescence collected at 1275 nm after excitation at 532 nm of air-equilibrated MB (blue) and MB-strep (red) solution. (**C**) Singlet oxygen emission intensity as a function of the absorbed energy for RB (magenta), TSPP (yellow), TMPyP (green), MB (blue), and MB-strep (red). Solid lines are the linear fits to the data.

The decrease in fluorescence yield observed for MB-strep raises the question of whether the formation of the MB triplet state is also affected by protein conjugation. Figure 2A reports the triplet state decay kinetics after nanosecond laser excitation for air-equilibrated PBS solutions of MB and MB-strep, monitored by the ground state bleaching recovery at 633 nm.

The kinetics for triplet decay of MB are best described by an exponential relaxation with a lifetime *τ* = (1.76 ± 0.05) µs, a value consistent with that expected for a triplet state quenched by molecular oxygen in air-equilibrated buffered solutions at room temperature. The triplet lifetime becomes longer, τ = (4.8 ± 0.1) µs, for MB-strep, an indication that the triplet state is slightly protected from molecular oxygen dissolved in solution. Although the observed lower amplitude suggests a lower triplet yield, a reliable estimate of this parameter is difficult, due to the spectral change associated with interactions of the dye with the protein environment. Nevertheless, an estimate based on the absorbance change collected at 633 nm affords *Φ_T_* = 0.03 ± 0.02 for MB-strep upon excitation at 580 nm, a tenfold lower value than for MB in PBS (*Φ_T_* = 0.52 [47]). This decrease is similar to what is observed for the fluorescence quantum yield (*vide supra*).

Singlet oxygen formation and decay were measured using time-resolved near-infrared phosphorescence [13]. Figure 2B reports representative kinetics for air-equilibrated PBS solutions of MB and MB-strep, corresponding to the highest concentrations used. The kinetics for MB clearly show a rise and fall that can be well described by a double exponential relaxation of equal amplitudes and opposite signs. The retrieved lifetimes are *τ*_1_ = (2.5 ± 0.3) μs and *τ*_2_ = (3.1 ± 0.3) μs, attributable to singlet oxygen formation and decay, respectively. The small signal for MB-strep prevents a reliable estimate of the rising part of the transient. The best fit was obtained with a double exponential relaxation of equal amplitudes, opposite sign, and lifetimes *τ*_1_ = (3.5 ± 0.5) μs (rise) and *τ*_2_ = (8.4 ± 0.2) μs (decay), reasonably identified as singlet oxygen decay and formation, respectively.

Singlet oxygen quantum yields for MB and MB-strep were determined by a comparative method (Figure 2C), using as reference molecules Rose Bengal (*Φ_Δ_* = 0.75 [55]), 5,10,15,20-tetrakis-(4-methylpyridyl)-porphine (TMPyP, *Φ_Δ_* = 0.74 [55]), and 5,10,15,20-tetrakis-(4-sulfonatophenyl)-porphine (TSPP, *Φ_Δ_* = 0.62 [56]). From these estimates, we obtain average values of *Φ_Δ_* = 0.55 ± 0.02 for MB, in agreement with the literature [47], and *Φ_Δ_* = 0.056 ± 0.003 for MB-strep upon excitation at 532 nm, in keeping with the triplet yield of the compound reported above.

### 2.3. ConA Effectively Targets Bacteria

**Figure 3:**
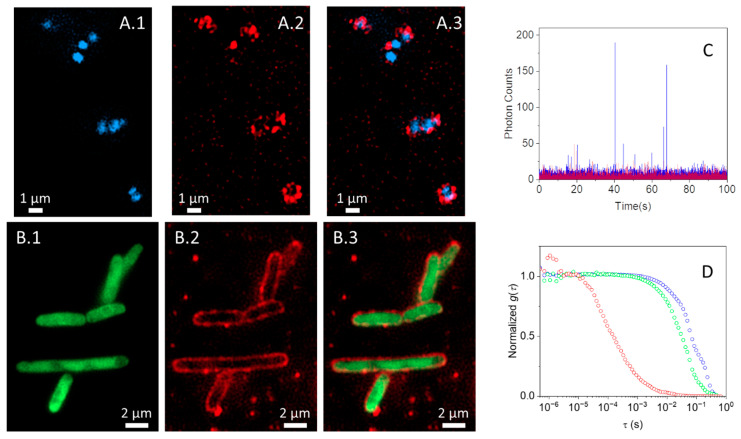
Fluorescence images *S. aureus* labeled with DAPI (blue channel, **A.1**) and *E. coli* expressing EGFP (green channel, **B.1**) exposed to ConA-STAR635 (red channel, **A.2** and **B.2**); merged channels are reported in (**A.3** and **B.3**). *S. aureus* and *E. coli* suspensions at OD_600_ ~ 0.5 were layered on a poly-L-lysine-treated 96-well glass bottom plate. Subsequently, bacteria were incubated with 1 µM ConA-STAR635 for one hour at room temperature. (**C**) Time traces collected for ConA-STAR635 in PBS at pH 7.4 (red) and in the presence of *E. coli* (blue). (**D**) Representative cross correlation curves for ConA-STAR635 in PBS at pH 7.4 (red, 0.1 μM) and for ConA-STAR635 (1 μM) in PBS at pH 7.4 in the presence of *S. aureus* (green) and *E. coli* (blue).

To check the targeting capability of ConA, we first directly labeled the protein at Lys residues with STAR635, a bright, red-emitting fluorophore, and collected fluorescence images of bacteria incubated with the compound. An *E. coli* strain expressing a green fluorescent protein was used to provide a reference for bacterial cytoplasm, whereas *S. aureus* was incubated with the blue-emitting DNA label DAPI to provide a reference for bacterial DNA. Because STAR635 and the green fluorescent protein (or DAPI) have spectrally well separated emission, they are simultaneously localized in the same field of view. Examples are given in Figure 3A (for *S. aureus*) and Figure 3B (for *E. coli*), where emission of ConA-STAR635 and the GFP (DAPI) are in red and green (blue), respectively.

For *E. coli*, emission by the cytoplasmic green fluorescent protein is surrounded by the red emission by ConA-STAR635 (Figure 3A), indicating that the compound has bound the bacterial wall extensively and is distributed quite uniformly. Unlike *E. coli*, ConA-STAR635 labeling of *S. aureus* is less uniform and incomplete, with a few bright red spots lining the blue emission by DAPI (Figure 3B), suggesting that the affinity for the constituents of the bacterial wall is lower in this case and that binding sites are distributed unevenly on the surface.

### 2.4. Fluorescence Correlation Spectroscopy Detects Binding of ConA to Bacteria

The binding of ConA-STAR635 is further confirmed by FCS experiments. The time traces in Figure 3C show that fluorescence fluctuations were observed for ConA-STAR635 in the absence of bacteria (red), become slower, with spikes of high intensity when bound to *E. coli* (blue), corresponding to bacteria entering the confocal volume. Similar traces are observed for *S. aureus*. The corresponding cross-correlation curves in Figure 3D are consistent with the observed binding. In particular, the cross-correlation curve for ConA-STAR635 is best described by a single diffusing species with diffusion coefficient *D* = 70 ± 10 μm^2^/s and a triplet contribution with lifetime *τ_T_* = 50 ± 10 μs. In the presence of bacteria, slower diffusing fluorophores appear, which we interpret as arising from ConA-STAR635 bound to bacteria.

We used the maximum entropy method (MEM) to recover the distribution of diffusion times underlying a given correlation curve. Of all distributions that fit the data with a given goodness of fit, e.g., chi-square, the MEM chooses the distribution possessing maximum entropy.

**Figure 4:**
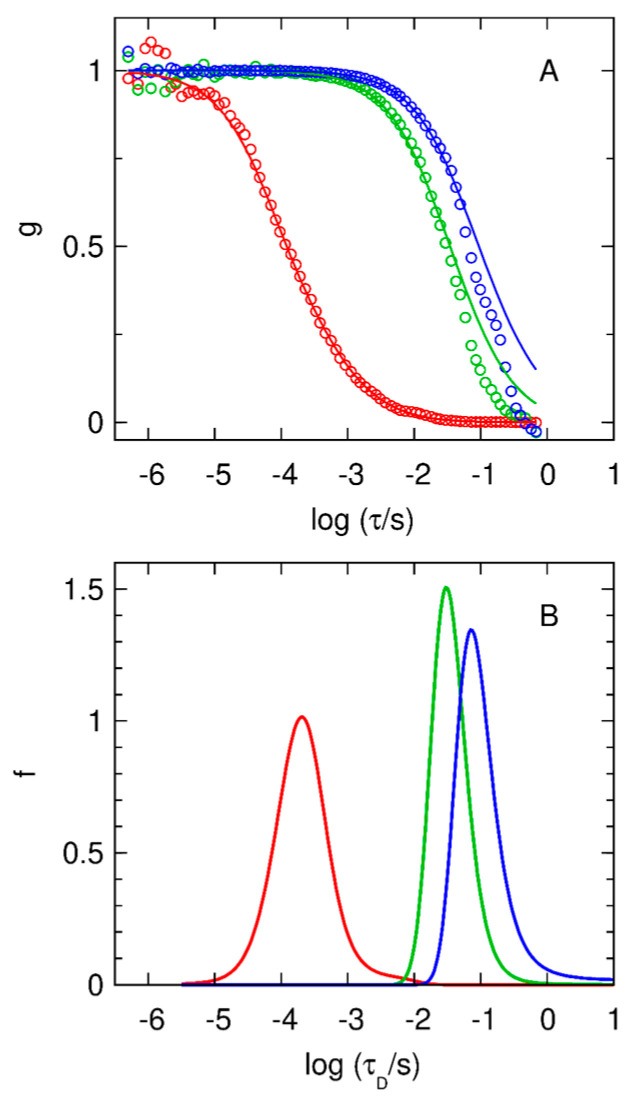
MEM analysis of the cross-correlation curves reported in Figure 3D. (**A**) Normalized cross-correlation curves for 0.1 μM ConA-STAR635 in PBS at pH 7.4 (red circles), and for 0.1 μM ConA-STAR635 in PBS at pH 7.4 in the presence of *S. aureus* (green circles) and *E. coli* (blue circles), and fits, g(log*τ*), obtained with the program MemDif (solid lines). For the fit to ConA-STAR635, the triplet-state lifetime was estimated to be 70 μs (*θ_T_* = 0.2). (**B**) Distributions of diffusion times, f(log *τ_D_*), corresponding to the fits in panel (A).

The distributions of diffusion times for ConA-STAR635 (Figure 4, red curve) include a peak centered at *τ_D_* ~ 4 × 10^−4^ s (*D* ~ 56 μm^2^/s), which we attribute to the diffusion of the labelled protein. The modeled curve includes a triplet state contribution with a time constant of *τ_T_* ~ 7 × 10^−5^ s. The *S. aureus* and *E. coli* data require single-band distributions with peaks at much longer diffusion times, with a mean *τ_D_* = 36 ms (*D* ~ 0.6 μm^2^/s, hydrodynamic radius *R_h_* ~ 0.3 μm) for *S. aureus* and mean *τ_D_* = 100 ms (*D* ~ 0.2 μm^2^/s, R_h_ ~ 0.9 μm) for *E. coli*. These slower peaks arise from proteins bound to bacteria. These fits deviate somewhat from the data at large τ, presumably due to the inadequate sampling of the infrequent motions of the bacteria in and out of the confocal volume. This limited sampling complicates the estimate of standard errors used in the MEM inversion, and therefore the distributions shown in Figure 4 were chosen conservatively. Fits by one discrete species (not shown) yield *τ_D_* = 32 ms for *S. aureus*, and *τ_D_* = 78 ms for *E. coli*. The triplet contribution is negligible in these curves.

### 2.5. ConA Drives the Full Supramolecular Construct to the Bacterial Wall

The full construct comprises the targeting unit (ConA biotinylated at the Lys residues; see Section 3) and the photosensitive unit (MB-strep). To demonstrate binding of ConA-strep to the bacterial wall, we first performed widefield imaging experiments on bacteria treated with the full construct, where MB was replaced by the brighter fluorophore STAR635. To observe binding of the compound to the bacterial wall, we were forced to use µM concentrations as previously explained for the imaging experiments with ConA-STAR635. This proved unfeasible when strep-STAR635 was used, due to the large background emission coming from strep-STAR635 molecules bound to the coverslip.

**Figure 5:**
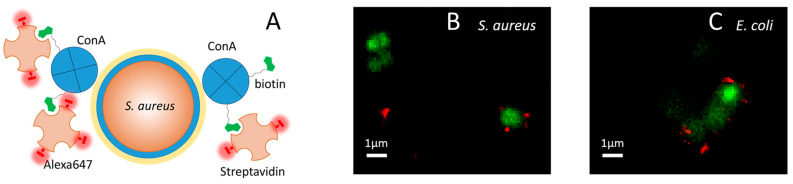
(**A**) Cartoon illustrating the structure of the fluorescent supramolecular construct ConA-Alexa647-strep. Biotin-(green)-labeled ConA tetramers (blue) bind Alexa647-(red)-labeled streptavidin tetramers (light orange). The interaction between ConA and the sugars on the surface of the bacterial wall drives the construct to the cellular target, in this example, *S. aureus*. Panels (**B**,**C**) Colocalization of bacterial DNA (green, widefield) and the ConA-Alexa647-strep construct (red, dSTORM) for *S. aureus* (**B**) and *E. coli* (**C**). For Syto13, excitation was at 488 nm; detection in the green channel was at 498–551 nm. For Alexa647, excitation was at 640 nm; detection in the red channel was at 685/40 nm.

We therefore performed a different type of imaging experiment, where strep was labeled with Alexa647 (Alexa647-strep), a fluorophore amenable to dSTORM microscopy. The supramolecular compound is sketched in Figure 5A, along with the interaction with the bacterial wall.

Figure 5 shows selected images of *S. aureus* (B) and *E. coli* (C), where the bacterial DNA is shown at low resolution in green, together with the dSTORM image of ConA-strep labeled with Alexa647 (ConA-Alexa647-strep, red). Thanks to the sub-diffraction spatial resolution provided by dSTORM [57], it is possible to appreciate that the red emission is located in just a few discrete spots of a few hundreds of nm, indicating that at the employed concentrations, the number of bound copies is limited by the moderate affinity of ConA for the saccharides present on the bacterial wall. Nevertheless, the red spots appear to decorate the outside of the bacterial wall, in keeping with expectations. The close proximity to the bacterial structure of the supramolecular compound confirms the fulfillment of the stringent localization requirements discussed in the introduction that are considered necessary to obtain an effective photosensitizer.

### 2.6. Photoinactivation of Bacteria

**Figure 6:**
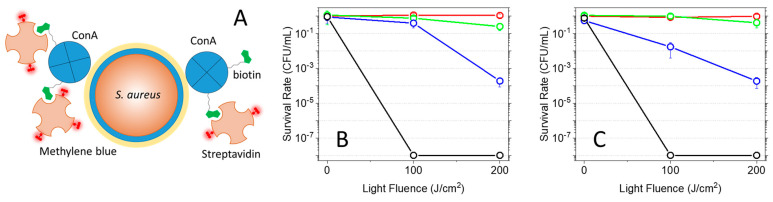
(**A**) Cartoon illustrating the structure of the photosensitizing supramolecular construct ConA-MB-strep. Biotin-(green)-labeled ConA tetramers (blue) bind MB-(dark red)-labeled streptavidin tetramers (light orange). The interaction between ConA and the saccharides on the surface of the bacterial wall drives the construct to the cellular target, in this example, *S. aureus*, where photosensitization of reactive oxygen species occurs. Panels (**B**,**C**) Light-fluence-dependent PDI of *S. aureus* and *E. coli*. (**B**) Plot of the CFU/mL survival fraction as a function of light-fluence for *S. aureus* treated with 5 μM ConA-MB-strep (blue circles), 5 μM MB (black), 5 μM MB-strep (green), and untreated (red). (**C**) Plot of the CFU/mL survival fraction as a function of light fluence for *E. coli* treated with 5 μM ConA-MB-strep (blue circles), 5 μM MB (black), 5 μM MB-strep (green), and untreated (red). Illumination was obtained with a red lamp (660 ± 10 nm).

The efficacy of the supramolecular construct ConA-MB-strep against bacteria was tested through in vitro photoinactivation assays on two model strains, the Gram-positive *Staphylococcus aureus* (Figure 6A) and the Gram-negative *Escherichia coli* (Figure 6B). Bacteria were first incubated with biotinylated ConA (5 μM) for 1 h to allow binding of the protein to the saccharides of the bacterial wall, and the suspension was then exposed to MB-strep (5 μM) for 30 min before irradiation with fluences of 100 J/cm^2^ and 200 J/cm^2^.

The plots reported below show that for both *S. aureus* and *E. coli*, a reduction in CFU/mL correlates with light fluence. In particular, a 4-log decrease is observed for bacteria treated with ConA-MB-strep and exposed to a 200 J/cm^2^ fluence. The lower photodynamic effect observed at 100 J/cm^2^ fluence for *S. aureus* in comparison with *E. coli* is consistent with the lower binding of ConA to the Gram-positive strain, asevidenced in the imaging experiments reported in Figure 3. Importantly, the green plots show that MB-strep leads to negligible phototoxicity and highlight the targeting capability of the construct when the targeting block ConA is bound to MB-strep. Thus, the assembly of the full supramolecular construct, combined with light activation, is necessary to obtain microbial inactivation.

The well-known phototoxicity of MB against bacteria [58,59,60] is evident in the control experiments reported in both plots, showing that exposure to a 200 J/cm^2^ fluence leads to an 8-log decrease in CFU/mL.

The lack of any effect of light exposure on untreated bacteria supports the absence of light-only photoinactivation.

The lower activity of the construct as compared to free MB is consistent with its lower triplet and singlet oxygen quantum yields and may reflect the moderate affinity of the construct for sugar molecules exposed to the bacterial wall. Localization of the construct may contribute to the observed efficiency since MB is known to penetrate the bacterial wall and bind to nucleic acids [61], whereas the supramolecular construct is restricted to binding to the bacterial wall, as evidenced in this work by fluorescence microscopy. Although the full photoactive construct appears to have lower photodynamic efficiency than MB alone, the capability of the ConA portion to bind exposed mannose and/or glucose groups is expected to confer an additional advantage in terms of antimicrobial activity by inhibiting bacterial adherence, the initial step in the development of diseases such as infectious keratitis [62]. Bacterial adherence inhibition by ConA treatment was reported in several cases [62,63,64,65,66]. For instance, inhibition of *E. coli* adherence to uroepithelial cells by ConA was obtained by pre-incubating bacteria prior to infection [65]. Importantly, while inhibitory effects against infection were reported for cells treated with sugars [65], the sugar concentrations necessary to induce a 50% inhibition were reportedly higher by orders of magnitude than those needed for ConA inhibition [65].

We finally wish to emphasize that the modular approach we have adopted gives high-functional flexibility through easy interchange between different photofunctional streptavidin units, either bearing bright fluorophores for high-resolution fluorescence imaging, or carrying photosensitizers to exert a phototoxic action against targeted bacteria. Nevertheless, the large mass of the compound may admittedly contribute to a reduction in observed affinity for saccharides, thus resulting in a lower photodynamic efficiency. Indeed, simpler and smaller compounds have shown higher photoinactivation efficiency, e.g., as documented by the study on ConA directly labeled with Rose Bengal [38].

## 3. Materials and Methods

Concanavalin A from *Canavalia ensiformis* (Jack bean), streptavidin from *Streptomyces avidinii* (salt-free, lyophilized powder), and methylene blue were from Sigma-Aldrich. The water-soluble Long-arm Biotin Labeling Kit was from MyBioSource (San Diego, CA, USA). STAR635 labelled strep and STAR635 NSH ester were from Abberior GmbH (Göttingen, Germany). Alexa647 NSH ester was from Invitrogen. Methylene-blue-labeled strep was from Atto-tech GmbH.

Concentrations were estimated from the molar absorption coefficients of the compounds: ε(280 nm) = 165,304 cm^−1^M^−1^ for streptavidin; ε(280 nm) = 116,000 M^−1^cm^−1^ for concanavalin A; ε(663 nm) = 85,000 M^−1^cm^−1^ for (monomeric) methylene blue; ε(635 nm) = 110,000 M^−1^cm^−1^ for STAR/635.

PD Minitrap™ G-25 columns were from Cytiva (Marlborough, MA, USA).

Both the biotinylation of Concanavalin A with the biotin labeling kit and the labeling with STAR635 exploit the reaction between an NHS ester group on biotin or the dye and the amine group of Lys residues on the protein. The reactions were performed following the suggestions of manufacturers. Briefly, the protein was incubated with a 5–10× excess of biotin or dye under slightly alkaline conditions and then purified by filtration or with a gel filtration gravity column. Absorption spectra gave a degree of labeling of up to two dyes per protein. Given the similarity of the labeling reactions, a comparable degree of labeling is estimated for biotin.

### 3.1. Laser Flash Photolysis

Triplet state decays of MB and MB-strep were monitored at 633 nm after photoexcitation with the 580 nm output of an OPO (GWU) pumped by the third harmonic (355 nm) of a nanosecond Nd:YAG laser (Surelite I-10, Continuum, San Jose, CA, USA) using a previously described setup [67,68]. Triplet quantum yields were estimated from laser flash photolysis using MB in PBS as a reference compound (*Φ_T_* = 0.52 [47]).

### 3.2. Fluorescence Quantum Yield

Absorption spectra were collected using a Jasco V-650 spectrophotometer (Jasco Europe, Cremella, Italy). Steady-state fluorescence emission spectra were collected using an SF5 spectrofluorometer equipped with temperature control, excitation, and emission polarizers (Edinburgh Instruments Ltd., Livingston, UK).

The fluorescence quantum yield $\Phi_{F}$ of MB-strep was calculated from the emission spectra using as reference molecule MB ($\Phi_{F}$ = 0.04 [47,53]):

(1) $$\Phi_{F,sample}=\Phi_{F,ref}*\frac{n_{sample}}{n_{ref}}*\frac{F_{sample}/\left(1-10^{-{A_{Ex}}_{sample}}\right)}{F_{ref}/\left(1-10^{-{A_{Ex}}_{ref}}\right)}$$

where n is the refractive index of the solvent, F is the area under the fluorescence emission curve, and $A_{Ex}$ is the absorbance at the excitation wavelength.

### 3.3. Singlet Oxygen Quantum Yields

Singlet oxygen (^1^O_2_) quantum yields (*Φ_Δ_*) were determined by time-resolved near-infrared phosphorescence using a customized Fluotime 200 time-resolved spectrophotometer (PicoQuant, Berlin, Germany). [13,69]. A diode-pumped Nd:YAG laser (FTSS355-Q, Crystal Laser, Berlin, Germany) working at a 10-kHz repetition rate (1.2 mJ per pulse, 1-ns pulse-width) was used for excitation at 532 nm. A 1064 nm rugate notch filter (Edmund Optics, Barrington, NJ, USA) and an uncoated SKG-5 filter (CVI Laser Corporation, Albuquerque, NM, USA) were placed in the laser path to remove any residual NIR emission. The ^1^O_2_ emission emerging from the sample was passed through a 1100 nm long-pass filter (Edmund Optics) and a narrow-bandpass filter centered at 1275 nm (BK-1270-70-B, bk Interferenzoptik) and was detected in a time-resolved manner using a thermoelectrically cooled NIR-sensitive photomultiplier tube assembly (H10330C-45-C3, Hamamatsu Photonics, Hamamatsu, Japan) and a multichannel scaler (NanoHarp 250, PicoQuant, Berlin, Germany). The time-dependent ^1^O_2_ phosphorescence intensity *S*(*t*) was then fitted by Equation (2), where *τ_T_* and *τ_Δ_* are the lifetimes of the photosensitizer triplet state and of ^1^O_2_, respectively, and *S*(0) is a preexponential parameter proportional to *Φ_Δ_*.

(2) St=S0×τΔτΔ−τT×(e−tτΔ−e−tτT)

*S*(0) values were determined at several sample absorbances to construct *S*(0) vs. $\left(1-10^{-A}\right)$ plots. The *Φ_Δ_* values were determined by comparing the slopes of such plots for the samples and suitable standards, as described by Equation (3):

(3) $$\phi_{\Delta ,sample}=\phi_{\Delta ,ref}\times \frac{{slope}_{sample}}{{slope}_{ref}}$$

The standards used were Rose Bengal (*Φ_Δ_* = 0.75 [55]), 5,10,15,20-tetrakis-(4-methylpyridyl)-porphine (TMPyP, *Φ_Δ_* = 0.74 [55]) and 5,10,15,20-tetrakis-(4-sulfonatophenyl)-porphine (TSPP, *Φ_Δ_* = 0.62 [70]).

### 3.4. Fluorescence Correlation Spectroscopy

FCS experiments were performed using a Microtime 200 system from PicoQuant (Berlin, Germany), based on an inverted confocal microscope and equipped with two SPADs (Single Photon Avalanche Diodes) used in cross-correlation mode. Excitation was achieved by a 635 nm picosecond diode laser operated at 20 MHz. Fluorescence emission by STAR635 or Alexa647 was collected through a bandpass filter (670/20 nm) and split with a 50/50 splitter between the two detection channels.

*S. aureus* and *E. coli* suspensions were washed with PBS to remove the culture medium from the solution. Subsequently, bacteria (OD_600_ ~ 0.2) were incubated with STAR635-labeled ConA (1 µM) for one hour at room temperature.

### 3.5. Analysis of Cross-Correlation Curves Using the Maximum Entropy Method (MEM)

Fluctuations $\delta F\left(t\right)=F\left(t\right)-\langle F(t)\rangle$ in fluorescence emission intensity F(t) by the excited molecules, collected from the confocal volume in the FCS setup, were analyzed by temporally autocorrelating δF(t) [71,72]:

$$G\left(\tau \right)=\frac{\langle \delta F\left(t\right)\delta F\left(t+\tau \right)\rangle }{\langle {F\left(t\right)}^{2}\rangle },$$

where $\langle F\left(t\right)\rangle =\frac{1}{T}\int_{0}^{T}F\left(t\right)dt$.

Correlation functions were analyzed in terms of distributed diffusion times *τ_D_* using the program MemDif. Written for this work, MemDif was adapted from MemExp [73], which has proven very useful in recovering distributions of lifetimes from noisy kinetics data [74,75,76]. MemDif and MemExp are available online.

Recently, the MEM was applied to fluorescence correlation spectroscopy in the context of a 2D Brownian diffusion model [77]. Here, the correlation-function data *G_i_* obtained at delay times *τ_i_* are fit by the expression [78]:

$$g_{i}=\frac{1-\theta_{T}+\theta_{T\;}e^{{-\tau }_{i}/\tau_{T}}}{1-\theta_{T}}\;\int_{-\infty }^{\infty }d\log\tau_{D}\;f(\log{\;\tau }_{D}){\left(1+\frac{\tau_{i}}{\tau_{D}}\;\right)}^{-1}{\left(1+\frac{\tau_{i}}{{\gamma^{2}\tau }_{D}}\right)}^{-1/2},$$

where *θ_T_* and *τ_T_* are the fraction of molecules in the triplet state and the state’s lifetime, respectively, and γ is the ratio of the major and minor axes of the ellipsoidal confocal volume. Here, γ = 6. The f distribution was recovered for given values of *θ_T_* and *τ_T_* by maximizing the entropy [79]

$$S\left(f,F\right)=-\sum_{j=1}^{M}[\;f_{j\;}\ln\left(\frac{f_{j}}{F_{j}}\right)+F_{j}-f_{j}]$$

while minimizing the value of chi-square:

$$\chi^{2}\left(G,g\right)=\frac{1}{N}\sum_{i=1}^{N}{\left(\frac{G_{i}-g_{i}\;}{\sigma_{i}}\right)}^{2}$$

The standard errors in the mean, *σ_i_*, were estimated from the deviations of a preliminary MEM fit from the data. The prior model ***F*** was taken to be uniform (a constant); it is the distribution recovered in the limit of zero signal-to-noise (no data).

When correcting for the triplet state, the simplex method was used to minimize *χ*^2^ with respect to *θ_T_* and *τ_T_*, with each evaluation of ***g*** invoking a MEM run with fixed values of *θ_T_* and *τ_T_*. The triplet-state correction was used only for the data taken in the absence of bacteria; for fits to data taken in the presence of bacteria, *θ_T_* was set to 0. The MemDif program was successfully tested on data simulated with and without triplet-state kinetics.

### 3.6. Widefield Fluorescence Imaging

Fluorescence imaging was performed with the motorized Axio Observer Inverted Fluorescence Microscope equipped with a Colibri 5/7 light source and an Axiocam 305 Mono using a 100×/1.3 NA oil-immersion objective and ZEN 3.1 blue software (ZEISS, Jena, Germany). EGFP (488/507) and STAR635 (635/655) were acquired to image *E. coli*, while DAPI (358/461) and STAR635 (635/655) were acquired to image *S. aureus*. A 4.76 μm Z-stack (18 slices with a 0.28 μm interval) was acquired, and a maximum-intensity projection of the Z-stack was produced to observe *S. aureus*-ConA binding. The images presented are acquisition files deconvolved using the regularized inverse filter deconvolution method in ZEN 3.1 blue software (ZEISS). 

*E. coli* DH5-α strain carrying the mEGFP-pBAD plasmid and *S. aureus* ATCC 29213 strain were inoculated in 3 mL of Luria–Bertani Miller broth (LBM) and incubated overnight at 37 °C in orbital shacking at 180 rpm to reach OD_600nm_ ≥ 1. Then, overnight cultures were diluted 1:100 in 3 mL of fresh medium and incubated for about 4 h at 37 °C in orbital shaking at 180 rpm until an OD_600nm_ between 0.5 and 0.6 was reached. To fluorescently label *E. coli* cells, mEGFP expression was induced by adding L-arabinose 0.15 M to culture medium at 2 h post-incubation. Then, bacterial cultures were dispensed at 40 μL/well in a 96-well imaging plate (Cellvis) pre-treated with 50 μL of 0.01% poly-L-Lysine (Merck, Darmstadt, Germany) and incubated for 30 min at room temperature to permit bacterial cell adhesion. Before the incubation for adhesion, 100 µL of *S. aureus* culture were pelleted by centrifugation (4000× *g* for 5 min) and resuspended in 1 mL of DAPI 15 μg/mL PBS. Then, 40 μL/well of cell suspension were dispensed to fluorescently label cells with DAPI during adhesion. Then, adhered bacteria were washed with PBS and exposed to 50 μL of ConA-STAR635 at 1 μM for 1 h at room temperature. Finally, samples were washed with PBS, and 50 μL/well of PBS were added for fluorescence imaging.

### 3.7. dSTORM Fluorescence Imaging

Fluorescence images of bacteria were collected with an ONI Nanoimager-S Mark III using a 100×, 1.45 NA oil immersion objective from Olympus. Fluorescence was simultaneously recorded in the blue-green (498–551 nm) and red (685/40) channels of the split sCMOS camera. Fluorescence excitation of Syto13 was obtained with a 488 nm laser. Alexa647 in dSTORM experiments was excited by a 640 nm laser. The exposure time was set to 50 ms for wide-field images. dSTORM images were collected using a 10 ms exposure time and recording 5000 frames. Images were collected over a 50 µm × 80 µm field of view in TIRF geometry.

Vegetative BL21 *E. coli* and ATC 25923 *S. aureus* cells were grown in sterile Tryptic Soy Broth at 37 °C until an optical density of 0.4 at 600 nm, then washed three times using PBS. Bacterial suspensions (approximately 40 μL) were placed in a chamber assembled from a glass slide and a coverslip (24 × 24 mm, thickness 0.15 mm) separated by a double-sided tape. To promote bacterial adhesion, clean coverslips were exposed to a poly-L-lysine 0.1 mg/mL solution for 30 min and then dried using nitrogen flow prior to incubation.

The bacterial suspension was transferred into the chamber and incubated with Syto13 for 30 min to provide a wide-field reference image of the bacterial DNA in the green detection channel. The sample was then incubated with biotinylated ConA (1 µM) for 1 h at room temperature on a shaker and washed with PBS. It was then incubated (for 30 min at room temperature on a shaker) with 0.2 µM (in tetramers) of Alexa647-labeled streptavidin, washed with 200 µL of STORM buffer (160 µL of PBS, 20 µL of MEA, 20 µL of glucose 5%, and 2 µL of GLOX), and eventually sealed.

### 3.8. Photoinactivation

Vegetative BL21 *E. coli* and ATC 25923 *S. aureus* cells were grown in sterile Tryptic Soy Broth at 37 °C until an optical density of 0.1 at 600 nm, corresponding to an initial concentration of bacteria of 10^6^ CFU/mL. Cell suspensions were then washed three times in PBS by means of centrifugation and resuspension. In one type of experiment, cells were first incubated in the dark with biotinylated ConA at 5 μM for 60 min at room temperature, then incubated for 30 more minutes with 5 μM MB-strep. In control experiments, cells were incubated for 30 min with either 5 μM MB-strep (in the absence of biotinylated ConA) or 5 μM MB.

Photoinactivation experiments were performed as previously described [80]. Suspensions were placed in 96-well plates and irradiated with red light using a LED light source (Red Light Man lamp) for which the red output at 660 ± 10 nm (48.3 mW/cm^2^) was selected.

Irradiation was performed with light fluences of 100 and 200 J/cm^2^. Irradiation alone (without exposure to the photoactive compounds) led to no appreciable effects on bacterial growth.

Suspensions were then serially diluted until 10^−6^ times the original concentration and then plated on Tryptic Soy agar plates. Colony-forming units (CFUs) were counted after 24 h incubation in the dark at 37 °C to calculate the survival fraction. Three independent assays were conducted, with six replicates within each assay. Survival fractions are expressed as means ± standard deviation.

## 4. Conclusions

In the exploration of molecular species capable of driving photosensitizers to bacterial targets in antimicrobial PDI, we have tested the efficacy of ConA as a targeting unit within the molecular platform we have proposed, based on the streptavidin-biotin binding properties. We have previously demonstrated the antimicrobial properties against *S. aureus* of the supramolecular photoactive complex formed between streptavidin functionalized with eosin and a biotinylated Immunoglobulin G [37]. The high affinity and selectivity of IgG towards *S. aureus* protein A resulted in selective targeting and highly efficient photokilling of these bacteria.

However, while protein–protein interactions occur with higher affinity and selectivity, it is expected that a selective pressure may lead to the development of resistance mechanisms where the targeted bacterial protein undergoes mutations that result in reduced affinity for the carrier protein. In this respect, the use of carrier proteins targeting saccharides on the bacterial wall may offer some advantages, as it could prove to be more robust against resistance emergence. Although this is a notable property, it should be emphasized that it is partly mitigated by the lower affinity of the carrier protein for the target, which reduces the load of photosensitizers on the bacterial wall and the subsequent photodynamic action. The latter effect also reflects the lower triplet and singlet oxygen yields of MB when embedded in the supramolecular complex. However, the inhibitory effect of ConA on bacterial adhesion is expected to exert a light-independent synergistic action that may improve the performance of the photoactive compound.

Nevertheless, a remarkable advantage of the proposed targeting system is its capability to address a wide spectrum of bacteria, both Gram-positive and Gram-negative. This result is significant, as it is quite unusual to find a single carrier construct that is effective against the two types of bacteria, Gram-negative bacteria being more difficult to photoinactivate, in general given the double membrane of their walls. Moreover, using the same approach, the construct may be specialized to hold any photosensitizer.

Finally, in this work, we also confirm the utility of Fluorescence Correlation Spectroscopy in the study of interactions between fluorescent substrates and large-size target structures. In this context, we analyze autocorrelation traces using a maximum entropy method, which proves particularly advantageous when describing signals measured for diffusing systems heterogeneous in size, such as fluorescent species bound to bacteria.

## Data Availability

The data presented in this study are available in this article.
